# Digital inclusive finance, industrial structure, and economic growth: An empirical analysis of Beijing-Tianjin-Hebei region in China

**DOI:** 10.1371/journal.pone.0299206

**Published:** 2024-03-19

**Authors:** Wenhai Zhou, Xiaoyu Zhang, Xiaomin Wu

**Affiliations:** 1 School of Economics, Hebei University, Baoding, China; 2 Center for Common Prosperity Research, Hebei University, Baoding, China; Universiti Malaysia Sabah, MALAYSIA

## Abstract

As a product of combining digital technology and traditional finance, digital inclusive finance plays a vital role in economic growth. This paper deeply analyzes the impact of digital inclusive finance on economic growth and the specific transmission path. This research selects the municipal panel data of Beijing-Tianjin-Hebei from 2011 to 2020 and empirically studies the impact of digital inclusive finance on economic growth. From the perspectives of industrial structure transformation speed, industrial structure upgrading, and industrial structure rationalization, this study analyzes the role of industrial structure in the impact of digital inclusive finance on economic growth and tests the heterogeneity of the impact of digital inclusive finance on economic growth. The results show that digital inclusive finance has a significant role in promoting economic growth. The depth of use of digital inclusive finance has the most significant impact, followed by the breadth of coverage, and the degree of digitization is the smallest. The industrial structure transformation speed and the industrial structure rationalization play a significant intermediary role in the economic growth effect of digital inclusive finance, and the industrial structure upgrading has no significant impact on the economic growth effect of digital inclusive finance; the promotion effect of digital inclusive finance on economic growth is bigger in the economically developed group, the higher digital inclusive finance group and the technologically developed group, and the promotion effect is smaller in the economically underdeveloped group, the lower digital inclusive finance group and the technologically underdeveloped group. The results provide a strong reference for policy formulation to promote the development of digital inclusive finance and economic growth.

## Introduction

Since the reform and opening up, China’s economic construction has made remarkable achievements. By the end of 2022, China’s GDP was as high as 121.02 trillion yuan, ranking the second largest economy in the world. This is inseparable from financial support. As the blood of the economy, finance has a huge role in promoting and regulating economic growth [1]. However, due to the limitation of time and space and the high cost of traditional finance, it is impossible to give full play to the supporting role of finance in economic growth [2]. How to find new economic growth points, optimize the industrial structure, and lead the new economic normal have become the focus of attention from all walks of life [3]. In this context, with the continuous impact of the digital wave, digital inclusive finance came into being.

Digital inclusive finance is a product of the deep integration of traditional finance and modern technologies such as the Internet, cloud computing, big data, and blockchain [3]; it is a technological revolution in traditional finance. Digital inclusive finance has the dual characteristics of digital technology and inclusive finance; it can get rid of the dependence of traditional finance on physical outlets, break the limitations of time and space [4], break through the service content and boundary of traditional finance [2], and can also be deeply integrated with the industry to promote industrial supply-side reform, improve production efficiency and optimize the allocation of production factors. It is regarded as an important engine to optimize the industrial structure. At the same time, digital inclusive finance has an impact on many fields such as innovation and entrepreneurship [2], consumption [5], savings [6], and poverty reduction [7], which in turn affect industrial structure and economic growth.

The Chinese government attaches great importance to the development of digital inclusive finance. The government work report of May 2020 affirmed the important role of digital inclusive finance in promoting economic development. The "Implementation Opinions on Promoting the High-quality Development of Inclusive Finance", adopted at the 24th Central Committee on February 28, 2022, emphasized accelerating the completion of financial services and promoting the rapid development of digital inclusive finance. According to the Peking University Digital Inclusive Finance Index [8], the average level of China’s digital inclusive finance index in 2011 was 40.00, and the digital inclusive finance index in 2020 was as high as 341.29. At the same time, as the largest urban agglomeration in northern China and the core growth pole of the country, the development level of digital inclusive finance is increasing daily. By 2020, the level of digital inclusive finance has reached 367.34, which is 26.05 higher than the average level of China’s digital inclusive finance in the same period. Whether the rapid development of digital inclusive finance also promote economic growth? In this context, the research on the impact of digital inclusive finance on economic growth has important practical significance and theoretical value for promoting sustained economic growth and leading the new normal of the economy. At the same time, in addition to theoretically answering whether digital inclusive finance contributes to economic growth, the following questions need to be solved: If digital inclusive finance can promote economic growth, what role does the industrial structure play in it? Is there a difference in the impact of digital inclusive finance on economic growth among the industrial structure transformation speed, industrial structure upgrading, and industrial structure rationalization? Due to the different economic levels, resource endowments, and development conditions of Beijing-Tianjin-Hebei cities, is the impact of digital inclusive finance on economic growth also different? Scientifically answering the above questions is significant for a comprehensive understanding of the relationship between digital inclusive finance, industrial structure, and economic growth.

This paper makes contributions in the following three aspects. (1) Based on the perspective of the Beijing-Tianjin-Hebei region, the largest urban agglomeration in northern China, this paper uses the data of prefecture-level cities to construct a panel model to explore the impact of digital inclusive finance on economic growth in Beijing-Tianjin-Hebei region and expands the relevant research on digital inclusive finance at the level of urban agglomeration. (2) This paper studies the role of industrial structure in the relationship between digital inclusive finance and economic growth from the perspectives of industrial structure transformation speed, industrial structure upgrading, and industrial structure rationalization. The empirical test of this transmission mechanism based on the intermediary model is helpful to fully understand the role of industrial structure in the effect of digital inclusive finance on economic growth. (3) According to the characteristics of economic growth, digital inclusive finance, and technological innovation, the heterogeneity of the impact of digital inclusive finance on economic growth is further investigated. These tests and explorations will help to expand the understanding of digital inclusive finance, industrial structure, and economic growth in theory and provide a theoretical basis for using digital inclusive financial tools to promote industrial structure development and stimulate economic growth.

The rest of this article is structured as follows. The literature review analyzes and summarizes the relevant literature. Mechanisms and research hypotheses present the theoretical mechanism and research hypothesis of digital inclusive finance, industrial structure, and economic growth. The research design introduces the regression method and data. Empirical results and discussion analyzes the regression results. Conclusions and policy implications summarize and make policy recommendations.

## Literature review

### Digital inclusive finance and economic growth

Most existing research believes that digital inclusive finance helps promote economic growth. Ali et al. [9], Beck et al. [10], and Lee et al. [3] conducted studies on E-7 economies, Kenya and China, respectively, all confirmed that digital inclusive finance helps promote economic growth. Kim et al. [11], Zhang et al. [12], and Zhang and Umair [13] pointed out that digital inclusive finance has the characteristics of fairness and efficiency, which can optimize the allocation of financial resources, help alleviate the resource curse, achieve economic recovery and promote economic growth. Wu & Wu [2] and Sun & Tang [14] pointed out that digital inclusive finance can help alleviate corporate financing constraints, enhance emergency response capabilities, improve corporate performance, and promote corporate development to stimulate economic growth. Li et al. [5], Ozili [15], and Zhang et al. [16] found that digital inclusive finance can help stimulate residents’ consumption and can also lead to residents’ green consumption, reduce environmental pollution, and achieve high-quality economic growth. However, some scholars believe that digital inclusive finance is not conducive to economic growth. Agwu [17], Philip & Williams [18], and Salemink et al. [19] pointed out that due to the slow development of rural digital inclusive finance, digital inclusive finance can only partially cover rural areas. The existence of the digital divide will increase the urban-rural income mismatch and may not be conducive to economic growth. However, in response to the problem of the digital divide, Liu & Guo [20] and Liu et al. [21] pointed out that with the continuous improvement of government facilities and the continuous development of digital inclusive finance, the digital divide has gradually narrowed, helping to alleviate relative poverty and reduce the income gap. Overall, digitally inclusive finance still helps promote economic growth.

### Digital inclusive finance and industrial structure

Existing research has confirmed that finance is an important path to promote the optimization of industrial structure, and it is generally believed that financial development helps to promote industrial structure upgrading [22]. Sasidharan et al. [23] and Pradhan et al. [24] believe that economic development can not only provide a good financial environment for the development of enterprises, meet the financing needs of enterprises, but also improve the level of financial resource allocation and help promote the upgrading of industrial structure. Bruhn & Love [25] and Chen & Zhang [26] pointed out that by integrating inclusive finance with digital technology, digital inclusive finance further expands financial coverage, improves financial coverage, optimizes credit investment structure, improves financial resource allocation level, and helps to promote industrial structure upgrading. In addition, scholars have also found that economic development encourages the development of scientific research and improves the level of enterprise innovation. Welter & Smallbone [27] and Zhang & Fan [28] found that digital inclusive finance makes up for the shortcomings of inclusive finance in supporting enterprise R&D and innovation, stimulates the enthusiasm for enterprise innovation, encourages enterprises to carry out scientific research and innovation actively, drives the transformation of industrial structure, and realizes the industrial structure upgrading. Li et al. [29] and Zhang et al. [16] found that digital inclusive finance has changed residents’ consumption habits, reshaped business models, promoted industrial transformation by changing consumer demand, and guided industrial structure optimization.

### Industrial structure and economic growth

Existing research generally confirms that industrial structure optimization helps to promote economic growth [30, 31]. Brandt et al. [32] and Rostow [33] believe that the essence of economic growth includes industrial structure optimization in addition to the growth of economic aggregate. The optimization of industrial structure means the transformation of production factors from low-efficiency sectors to high-efficiency sectors. Chen and Xie [34] and Qiu et al. [35] found that in the process of transforming production factors into high-efficiency sectors, the rationalization of industrial structure helps to improve the level of production technology and will also produce knowledge spillover effects, which will help drive the technological level of neighboring regions, enhance market efficiency, and have a significant role in promoting economic growth. Scholars have also found that the promotion effect of industrial structure optimization on economic growth is not linear. Shi [36] studies China’s coastal areas and finds that rationalizing industrial structure has a significant threshold effect on economic growth. When the level of economic development is high, the promotion effect of industrial structure rationalization on economic growth will be weakened. Zhao and Zhu [37] and Wu et al. [38] divided industrial structure adjustment into industrial structure rationalization and industrial structure upgrading from the perspective of industrial structure adjustment and found that industrial structure adjustment is helpful to rationally allocate production factors, accelerate the process of low productivity to high productivity transformation, to improve economic growth.

Although scholars have done a lot of research on digital inclusive finance, industrial structure, and economic growth, they generally study two of them at the same time and seldom include digital inclusive finance, industrial structure, and economic growth in the same analytical framework for analysis, ignoring the correlation between the three. The existing research is mostly analyzed from the rationalization or upgrading of industrial structure. However, the industrial structure can be divided into three levels: the industrial structure transformation speed, the industrial structure upgrading, and the industrial structure rationalization; the analysis of industrial structure is not comprehensive enough and needs to be further improved.

## Mechanisms and research hypotheses

### Digital inclusive finance and economic growth

First of all, digital inclusive finance endows traditional finance with information technology [39]. With the natural advantages of efficient search, calculation, and analysis of data, it realizes the interconnection of information [40], reduces the adverse selection problem caused by information asymmetry between the supply and demand sides of funds [41], improves the efficiency of financial services [42], optimizes the matching degree of financial capital and real assets, realizes the optimal allocation of financial resources, and gives full play to the significant role of finance in promoting economic growth [7]; Secondly, digital inclusive finance also expands financing channels [43], reduces the threshold of financial services and financing costs [7], provides more convenient financing services for enterprises and residents [44], effectively alleviates the financing constraints faced by enterprises, promotes enterprise innovation and industrial development [2, 9], and promotes macroeconomic growth. Thirdly, with its excellent universality and low threshold characteristics, digital inclusive finance has improved the availability of financial services [5, 7]. Groups marginalized by traditional financial services can obtain financial resources, broaden the coverage of financial services, and reduce ’financial exclusion’ [45]. It helps to break the restrictions of urban-rural dual economic structure, alleviate rural financial repression, improve rural financial availability, provide convenient, fast, equal and efficient financial services for rural and vulnerable groups [42], and promote economic growth [46]; Finally, with the help of digital service platform, digital inclusive finance not only enables residents to enjoy convenient payment channels, but also alleviates residents’ liquidity constraints through installments and consumer loans, uses inter-temporal consumption balance, releases residents’ potential consumption ability, and promotes residents’ consumption to drive economic growth [5]. Based on this, this paper proposes the first research hypothesis:

**Hypothesis 1:** Digital inclusive finance promotes economic growth.

### Digital inclusive finance, industry structure, and economic growth

The optimization of industrial structure helps to realize the optimal allocation of production factors under the limited natural endowment so that the production factors flow from low-efficiency departments to high-efficiency departments, promote the improvement of the level of productivity of the whole society, and promote economic growth [31]. First of all, digital inclusive finance expands the scope of financial coverage, provides venture capital for vulnerable groups and small, medium, and micro enterprises that are excluded from the scope of traditional financial services [47], and provides financial services for new formats and new industries, which is conducive to opening up new industries, accelerating the transformation of industrial structure [48], improving the efficiency of resource use and promoting economic growth. Secondly, digital inclusive finance expands the breadth and depth of financial services [49], broadens financing channels, invests more financial resources in high-tech industries, optimizes the distribution of funds in the industry, improves the efficiency of resource use, and provides financial resources for enterprise technological innovation to achieve green technology transformation [2], encourage enterprises to eliminate the original high-cost and high-consumption traditional industries, improve total factor productivity, promote industrial structure upgrading [4, 50], and promote economic growth. At the same time, digital inclusive finance improves the efficiency of financial resource allocation [25], reduces information asymmetry, realizes the rational allocation of production factors, avoids blind investment by enterprises, strengthens the degree of correlation between industries, realizes coordinated development between industries, improves the rationalization level of industrial structure, and drives economic growth. Finally, by realizing inter-temporal consumption, digital inclusive finance has increased the service group of consumer financing, stimulated consumption demand and structure [51], and made residents pay more attention to green consumption [14]. The change in consumption demand and structure has promoted industrial structure optimization and stimulated economic growth. Based on this, the following research hypotheses are proposed:

**Hypothesis 2:** The industrial structure transformation speed plays an intermediary role in the impact of digital inclusive finance on economic growth.
**Hypothesis 3:** The industrial structure upgrading plays an intermediary role in the impact of digital inclusive finance on economic growth.
**Hypothesis 4:** The industrial structure rationalization plays an intermediary role in the impact of digital inclusive finance on economic growth.

### Research design

**Fig 1:**
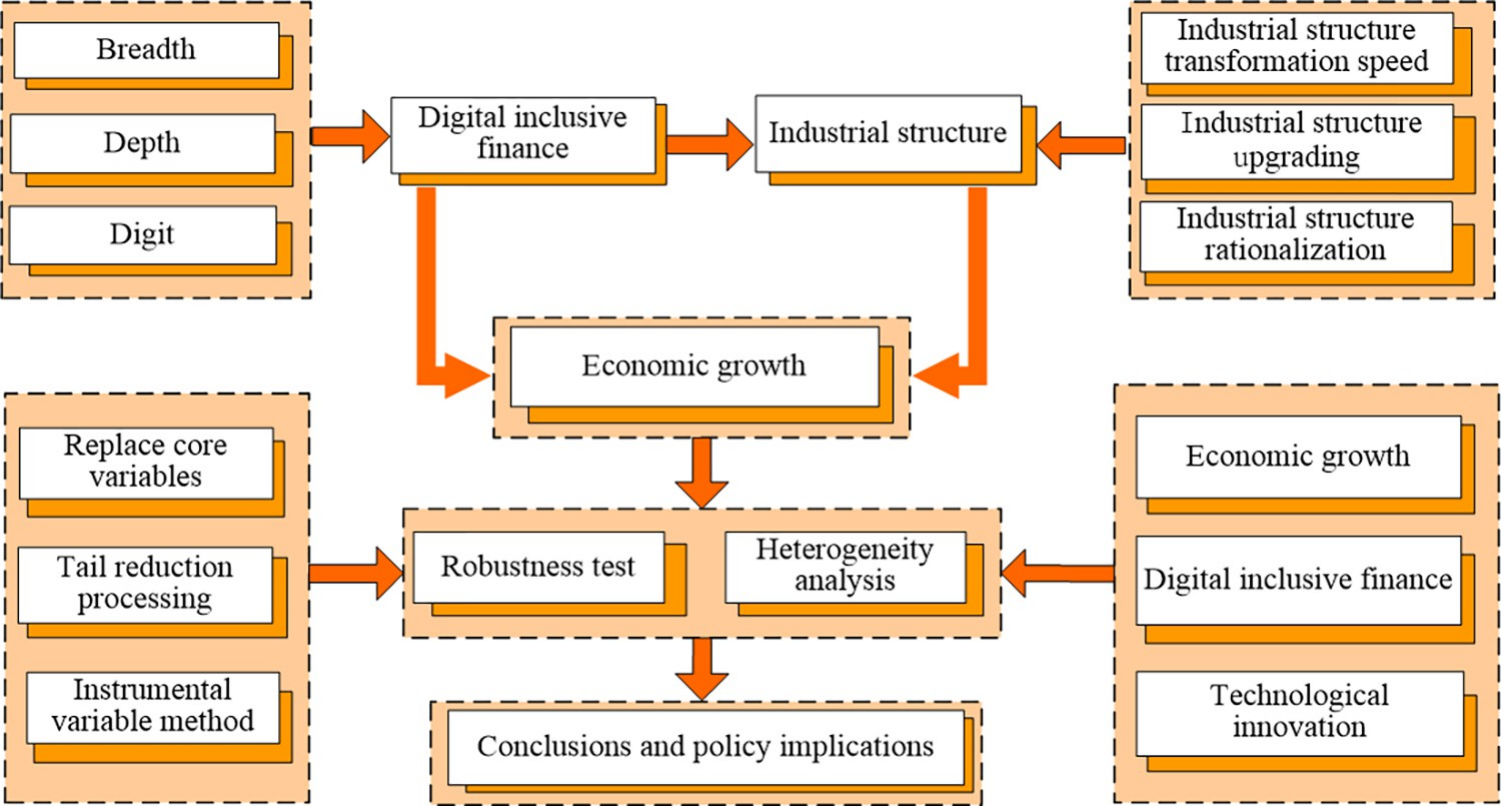
Article structure diagram.

This paper analyzes the direct impact of digital inclusive finance on economic growth and passes the robustness test. This research discusses the indirect effect of digital inclusive finance on economic growth from three perspectives: industrial structure transformation speed, the industrial structure upgrading, and the industrial structure rationalization. We also explore the heterogeneity of the economic growth effect of digital inclusive finance. The Article structure diagram is shown in **Fig 1**.

### Variable definition

#### Explained variable

Economic Growth (GDP). This index represents the regional economic development. Referring to the practice of Wang and Wen [52], the per capita GDP is selected to represent the level of economic growth. The larger the index, the higher the level of economic growth. Considering the impact of inflation, this paper takes 2010 as the base period and calculates the real per capita GDP of each city in 2011–2020 according to the GDP deflator.

#### Explain variable

Digital Inclusive Finance (DIF). This paper uses the Digital Financial Inclusion Index of Peking University [8]. It selects the total index of digital inclusive finance (DIF) and its three sub-dimensions of breadth of coverage (Breadth), depth of use (Depth), and degree of digitization (Digit). The larger the value of each index, the higher the development of digital inclusive finance.

#### Control variable

Based on previous research, we also select per capita capital (K), human capital (Edu), infrastructure construction (Ins), foreign investment (Open), and technological innovation (Patent) as control variables.

Per capita capital (K). It represents the degree of material capital accumulation. Referring to the practice of Zhao and Zhu [37], with 2010 as the base period, per capita capital is calculated by the perpetual inventory method. The larger the index, the higher the per capita capital and the more conducive to promoting economic growth.

Human capital (Edu). This indicator indicates the education level of the regional population, expressed by the number of college students per 10,000 people.

Infrastructure construction (Ins). The per capita urban road area is selected to measure the level of infrastructure construction.

Foreign investment (Open). Select the proportion of foreign direct investment in GDP to measure.

Technological innovation (Patent). The number of patent applications per capita is used to measure scientific and technological innovation.

#### Mediator variable

Industrial structure (Str). This paper selects the industrial structure transformation speed (Str1), industrial structure upgrading (Str2), and industrial structure rationalization (Str3) as three dimensions to measure industrial structure.

Industrial structure transformation speed (Str1). Referring to the practice of Ren et al. [39], the hierarchical coefficient of industrial structure is used, such as Formula (1), where y_1_, y_2_, and y_3_ represent the proportion of the output value of the primary, secondary, and tertiary industries. The larger the value, the faster the transformation of the industrial structure.

$$Str1=y_{1}\times 1+y_{2}\times 2+y_{3}\times 3$$ (1)

Industrial structure upgrading (Str2). Referring to the practice of Zhao & Zhu [37], the ratio of the output value of the tertiary and secondary industry is expressed, as shown in Formula (2), where Y_3_ and Y_2_ represent the output value of the tertiary industry and the secondary industry, respectively. The larger the value, it shows that the higher the level of industrial structure upgrading.

$$Str2=Y_{3}/Y_{2}$$ (2)

Industrial structure rationalization (Str3). As shown in Formula (3). Referring to the practice of Zhao & Zhu [37], this paper constructs the Theil index of industrial structure. In order to make the rationalization of industrial structure into a positive index, this paper chooses to use the (1-Theil index of industrial structure) to indicate the rationalization of industrial structure. The greater the value, the more reasonable the industrial structure. Among them, Y and Y_i_ represent the total output value and the output value of the I industry, respectively, and L and L_i_ represent the total employment number and the employment number of the I industry, respectively.


$$Str3=1‐\sum_{1}^{3}(\frac{Y_{i}}{Y})ln(\frac{Y_{i}/L_{i}}{Y/L})$$ (3)

The detailed description of each variable is shown in **Table 1**.

**Table 1:** Variable definition.

	Variable	Definition
Explained variable	GDP	Real per capita GDP
Explain variable	DIF	Peking University Digital Financial Inclusion Index
	Breadth	Peking University Digital Financial Inclusion Index of coverage breadth
	Depth	Peking University Digital Financial Inclusion Index of usage depth
	Digit	Peking University Digital Financial Inclusion Index of digitization
Control variable	K	The per capita capital is calculated by the perpetual inventory method
	Edu	Number of college students per 10,000
	Ins	Per capita urban road area
	Open	Foreign direct investment as a share of GDP
	Patent	Number of authorized patent applications per capita
Mediator variable	Str1	Hierarchical coefficient of industrial structure
	Str2	The ratio of tertiary industry to secondary industry output value
	Str3	1-Theil Index of Industrial Structure

### Methodology

#### Benchmark model

In order to study the impact of digital inclusive finance on economic growth in 13 cities of Beijing, Tianjin and Hebei, the following benchmark regression model is set by referring to the existing research [39], as shown in Formula (4).

$$LnGDP_{it}=\alpha_{0}+\alpha_{1}LnDIF_{it}+\alpha_{c}LnControl_{it}+\xi_{i}+\theta_{t}+\upsilon_{it}$$ (4)

Where LnGDP_it_ represents the economic growth level of city i in period t, α_0_ is the intercept term, α_1_ is the estimated coefficient of the core explanatory variable, DIF_it_ means the digital inclusive finance level of city i in period t, α_c_ is the estimated coefficient of the control variable, LnControl_it_ is a group of control variables, ξ_i_ represents the individual fixed effect, θ_t_ represents the time fixed effect, and υ_it_ means the random error term.

Mediation model. In order to test the impact mechanism of digital inclusive finance on economic growth, the following Mediation effect model is constructed, as shown in Formula (5).

$$LnStr_{it}=\beta_{0}+\beta_{1}LnDIF_{it}+\beta_{c}LnControl_{it}+\xi_{i}+\theta_{t}+\upsilon_{it}$$ (5)

In order to avoid endogenous bias, the Formula (5) is constructed by referring to the practice of Jiang [53]. Because the role of industrial structure in promoting economic growth is obvious, under the premise that Formula (4) digital inclusive finance has a significant effect on economic growth, if Formula (5) digital inclusive finance promotes industrial structure optimization, it shows that industrial structure has an intermediary role; if the impact of digital inclusive finance on industrial structure is not significant, then industrial structure has no intermediary role in the economic growth effect of digital inclusive finance. In the Formula (5), LnStr_it_ represents the level of industrial structure in the t period of i city, β_0_ is the intercept term, β_1_ is the core explanatory variable to be estimated coefficient, β_c_ is the control variable to be estimated coefficient, and the remaining variables are the same as above.

## Empirical results and discussion

### Data sources and descriptive statistics

Since the data of the Peking University Digital Inclusive Financial Index began in 2011, considering the availability of data, this paper selects the panel data of 13 cities in Beijing, Tianjin, and Hebei from 2011 to 2020 for research. Among them, the digital inclusive financial data comes from the Peking University Digital Inclusive Financial Index, and the remaining data comes from the Hebei Economic Yearbook, the China City Statistical Yearbook, the National Bureau of Statistics, and the Wind database. In order to smooth the data and eliminate the influence of heteroscedasticity, this paper takes the logarithm of all variables, and the descriptive statistics are shown in **Table 2**.

**Table 2:** Descriptive statistics.

Variable	N	Mean	S.D.	Min	Max
LnGDP	130	10.724	0.447	9.850	11.765
LnDIF	130	5.062	0.495	3.676	5.743
LnBreadth	130	5.010	0.520	3.350	5.731
LnDepth	130	5.042	0.475	3.870	5.745
LnDigit	130	5.211	0.567	2.900	5.778
LnK	130	3.253	0.370	2.477	4.199
LnEdu	130	5.016	0.706	3.548	6.252
LnIns	130	1.235	0.562	0.254	2.536
LnOpen	130	0.488	0.691	-2.040	2.074
LnPatent	130	1.605	1.098	-0.999	4.309
LnStr1	130	0.857	0.071	0.741	1.042
LnStr2	130	0.098	0.550	-0.662	1.667
LnStr3	130	-0.424	0.288	-1.133	-0.001

### Multicollinearity diagnostics

Before the empirical analysis, it is necessary to test the multicollinearity of the variables involved. This paper chooses the variance inflation factor to test the multicollinearity. The test results are shown in **Table 3**. It can be seen from Table 3 that the variance inflation factors of each variable are strictly less than 5, indicating that there is no multicollinearity. Therefore, in the empirical analysis, the multicollinearity problem can be ignored.

**Table 3:** Variance inflation factor.

Variable	VIF	1/VIF
LnDIF	2.08	0.48
LnK	2.00	0.50
LnEdu	2.45	0.41
LnIns	3.17	0.32
LnOpen	2.22	0.45
LnPatent	3.69	0.27
Mean VIF	2.60	

### Benchmark regression results

In order to explore the impact of digital inclusive finance on economic growth, this paper selects digital inclusive finance (LnDIF) and three sub-dimensions of digital inclusive finance: coverage breadth (LnBreadth), use depth (LnDepth), and digitization degree (LnDigit) for research. The empirical results are shown in **Table 4**.

**Table 4:** Baseline regression results.

Variable	(1)	(2)	(3)	(4)
LnDIF	0.151***			
	(11.850)			
LnBreadth		0.125***		
		(8.782)		
LnDepth			0.156***	
			(10.131)	
LnDigit				0.094***
				(10.930)
LnK	0.542***	0.551***	0.495***	0.679***
	(7.837)	(6.778)	(7.279)	(12.066)
LnEdu	0.059**	0.040	0.072**	0.030
	(2.497)	(1.706)	(2.724)	(1.242)
LnIns	0.090*	0.085	0.079*	0.081*
	(1.807)	(1.764)	(1.964)	(1.869)
LnOpen	0.010	0.006	0.005	0.016
	(0.556)	(0.303)	(0.386)	(0.925)
LnPatent	0.051**	0.070**	0.063***	0.064***
	(2.565)	(2.808)	(4.814)	(3.323)
Cons	7.701***	7.884***	7.767***	7.660***
	(31.525)	(27.751)	(37.124)	(33.043)
N	130	130	130	130
R^2^	0.974	0.968	0.973	0.971

Note: ***, **, * refer to the statistics being significant at the 1%, 5%, and 10% levels, respectively.

Column (1) of **Table 4** estimates the total index of digital inclusive finance (LnDIF). From the perspective of core explanatory variables, the influence coefficient of the index of digital inclusive finance (LnDIF) on economic growth (LnGDP) is 0.151, which is significant at 1%, indicating that digital inclusive finance promotes economic growth. Hypothesis 1 has been preliminarily verified and is consistent with previous research results [14, 52]. This result may be because digital inclusive finance has the advantages of universality, convenience, and low cost. It promotes economic growth by reducing the credit threshold of enterprises, improving the efficiency of financial resource allocation, improving industrial productivity, broadening the scope of financial services, alleviating the liquidity constraints of residents, and stimulating residents’ consumption.

From the perspective of control variables, regression coefficients of per capita physical capital (LnK), human capital (LnEdu), infrastructure construction (LnIns), and technological innovation (LnPatent) are all significantly positive, mainly because the more physical capital and human capital accumulation, the more production factors of economic growth, the higher the productivity level and technological innovation ability, the more conducive to economic growth. Perfect infrastructure construction can accelerate regional technology exchange and flow of production factors, improve the external environment for regional development, enhance the ability to attract foreign investment, and promote regional economic growth. The regression coefficient of the level of foreign investment (LnOpen) to economic growth is insignificant, because in recent years, the proportion of foreign investment in high-tech industries has gradually declined. The total investment in high-tech industries is decreasing, so the employment and spillover effects of science and technology on economic growth are not noticeable.

Columns (2)—(4) of **Table 4** are the estimation results of the three sub-dimensions of digital inclusive financial coverage breadth (LnBreadth), depth of use (LnDepth), and degree of digitization (LnDigit). The results show that the coefficients are 0.125,0.156, and 0.094, respectively, and are all significant at the 1% level, indicating that the better the coverage breadth, depth of use, and digitization of digital inclusive finance, the more helpful it is to promote economic growth. Hypothesis 1 has been further verified. At the same time, comparing the regression coefficients of the three sub-dimensions, it is found that the depth of use has the greatest impact on economic growth, followed by the breadth of coverage, and the digitization degree is the smallest. Other control variables are basically the same as column (1). Since previous studies have paid more attention to the impact of the total index of digital inclusive finance on the economy and ignored the impact of the sub-dimensions of digital inclusive finance [12, 52, 54], this conclusion further enriches the existing research results.

### Robustness tests

#### Replace core explanatory variables

Replacing the core explanatory variables is a common robustness test method [55–57]. This paper draws on the practice of Zhao [58] and selects five indicators: Internet penetration rate, number of Internet-related employees, Internet-related output, number of mobile Internet users and digital inclusive financial development index. The principal component analysis method is used to measure the comprehensive development index of digital economy to replace digital inclusive finance for regression. The regression results are shown in the first column of **Table 5**. The comprehensive development index of digital economy significantly promotes economic growth, consistent with the benchmark regression results.

**Table 5:** Robustness test results.

Variable	(1)	(2)	(3)
LnDIF	0.036**	0.149***	0.143***
	(2.433)	(12.863)	(9.805)
LnK	0.725***	0.512***	0.440***
	(7.910)	(7.384)	(6.331)
LnEdu	-0.017	0.056**	0.050*
	(-0.613)	(2.578)	(1.587)
LnIns	0.035	0.105*	0.088**
	(0.971)	(1.983)	(2.466)
LnOpen	0.013	0.009	0.021*
	(0.786)	(0.392)	(1.966)
LnPatent	0.112***	0.056**	0.033**
	(4.357)	(2.232)	(1.793)
Cons	8.222***	7.797***	7.720***
	(31.469)	(31.950)	(30.266)
Kleibergen-Paap rk LM			27.99
Kleibergen-Paap Wald rk F			115.54
N	130	130	130
R^2^	0.812	0.764	0.962

Note: ***, **, * refer to the statistics being significant at the 1%, 5%, and 10% levels, respectively.

#### Tail reduction processing

In order to prevent the influence of sample outliers on the research results, this paper conducts a 1% tail reduction on all sample data, including control variables. The second column of **Table 5** is the test results of sample tail reduction. The regression results do not change significantly. The regression coefficient of digital inclusive finance is consistent with the benchmark regression results, indicating that the benchmark regression results are robust and credible.

#### Instrumental variable method

Due to the endogenous problems that digital inclusive finance and economic growth may cause each other, this paper selected the number of fixed-line telephones at the city level in 1984 and the number of Internet users nationwide in the previous year as instrumental variables [58] and uses the two-stage least squares method (IV-2SLS) to estimate. This is because the telecommunications infrastructure can affect the application of subsequent Internet technologies, but the impact of fixed-line telephones on economic growth is not obvious. At the same time, in order to be able to use panel data for analysis, this paper draws on the Nunn & Qian calculation method [59]. It introduces the national Internet population that changes over time to construct instrumental variables. The regression results are shown in Column 3 of **Table 5**. The digital inclusive finance regression coefficient is still significantly positive, indicating that the benchmark regression results are robust and credible.

### Mechanism analysis

From the previous analysis, digital inclusive finance is conducive to the optimization of industrial structure, and the optimization of industrial structure plays a vital role in promoting economic growth. Therefore, there may be a transmission mechanism of "digital inclusive finance-industrial structure-economic growth". In order to verify this transmission mechanism, this paper examines the impact of digital inclusive finance on economic growth from three aspects: industrial structure transformation speed (LnStr1), industrial structure upgrading (LnStr2), and industrial structure rationalization (LnStr3). The regression results are shown in **Table 6**.

**Table 6:** Mechanism analysis results.

Variable	(1)	(2)	(3)	(4)
	LnGDP	LnStr1	LnStr2	LnStr3
LnDIF	0.151***	0.047*	0.166	0.509*
	(11.850)	(1.666)	(0.428)	(1.869)
Cons	7.701***	0.264**	-3.066*	-2.577**
	(31.525)	(2.215)	(-1.931)	(-2.229)
control	YES	YES	YES	YES
N	130	130	130	130
R^2^	0.974	0.554	0.471	0.129

Note: ***, **, * refer to the statistics being significant at the 1%, 5%, and 10% levels, respectively.

In order to facilitate the analysis, we still show the regression results of digital inclusive finance on economic growth in Column (1) of Table 6. Columns (2) — (4) are the regression results of digital inclusive finance on the industrial structure transformation speed, upgrading, and rationalization. Table 6 shows that digital inclusive finance has a positive effect on the industrial structure transformation speed and the industrial structure rationalization at the level of 10%. Still, digital inclusive finance has no obvious impact on industrial structure upgrading. This shows that based on digital inclusive finance, it significantly promotes economic growth; the industrial structure transformation speed and the industrial structure rationalization play an intermediary role in digital inclusive financial and economic growth. Still, the industrial structure upgrading does not play an intermediary role. Hypothesis 2 and Hypothesis 4 are verified. Most of the existing studies focus on upgrading industrial structure, ignoring the role of industrial structure transformation speed and rationalization, and the analysis of industrial structure is one-sided [16, 60]. This paper analyzes the impact of industrial structure from three perspectives: industrial structure transformation speed, industrial structure upgrading, and industrial structure rationalization, which further enriches the existing research results. Digital Inclusive Finance broadens financial coverage, reduces financial exclusion, provides financial services for new industries, improves the efficiency of resource allocation, realizes the coordinated development of industries, speeds up the transformation of industrial structure, and improves the level of industrial structure rationalization, thus promoting economic growth. The industrial structure upgrading does not play an intermediary role in the economic growth of digital inclusive finance. This may be because the construction of digital inclusive finance in some areas of Beijing, Tianjin, and Hebei is not yet perfect, and the role of digital inclusive finance in promoting the tertiary industry has not been fully played, resulting in the intermediary effect of digital inclusive finance on the industrial structure upgrading is not significant.

### Heterogeneity analysis

In order to further study the heterogeneity of the impact of digital inclusive finance on economic growth, this paper divides them into two groups according to the medians of economic growth (LnGDP), digital inclusive finance (LnDIF), and technological innovation (LnPatent) of 10.680,5.214 and 1.422. Columns (1), (3), and (5) indicate that the indicators are less than the median, and columns (2), (4), and (6) suggest that the indicators are greater than or equal to the median. The specific estimation results are shown in **Table 7**.

**Table 7:** Heterogeneity analysis results.

Variable	(1)	(2)	(3)	(4)	(5)	(6)
LnDIF	0.129***	0.206***	0.149***	0.474***	0.125***	0.260***
	(19.457)	(6.420)	(17.322)	(6.635)	(12.282)	(8.418)
Cons	7.659***	7.757***	7.348***	6.250***	7.591***	6.852***
	(41.283)	(15.559)	(47.431)	(13.497)	(57.185)	(28.713)
Control	Yes	Yes	Yes	Yes	Yes	Yes
N	65	65	65	65	65	65
R^2^	0.770	0.289	0.733	0.651	0.746	0.510

Note: ***, **, * refer to the statistics being significant at the 1%, 5%, and 10% levels, respectively.

From **Table 7**, it can be found that the regression coefficients of digital inclusive finance in each column are significantly positive at the 1% level. Still, there are some differences in the coefficients, indicating that there is heterogeneity in the role of digital inclusive finance in promoting economic growth.

From the perspective of the economic growth (LnGDP), the regression coefficient of the economically underdeveloped group is 0.129, indicating that for every 1% increase in digital inclusive finance, economic growth is 0.129%, while the regression coefficient of the economically developed group is 0.206. Its role in promoting the economy is more significant than that of the financially underdeveloped group. This result may be because compared with the economically developed group and the economically underdeveloped group, the financial institutions, the Internet, and other infrastructure construction are more perfect, the level of financial technology development is higher, the coverage of digital inclusive finance is wider, and digital inclusive finance can better serve economic growth.

From the perspective of digital inclusive finance (LnDIF), the regression coefficients of the lower and higher groups of digital inclusive financial development level are 0.149 and 0.474, respectively, indicating that the higher group of digital inclusive financial development has a more significant role in promoting economic growth. This result may be because the higher group of digital inclusive financial development generally has more financial institutions, financial products, and financial talents, residents have higher use of digital inclusive finance, and digital inclusive finance has a better effect on economic growth.

From the perspective of technological innovation (LnPatent), the regression coefficients of the technologically underdeveloped group and the developed group are 0.125 and 0.260, respectively, indicating that digital inclusive finance could better promote economic growth under the conditions of technological development. This result may be because the higher level of technological innovation helps to optimize the data algorithm of digital inclusive finance, improve product performance, improve the operational efficiency and coverage of digital inclusive finance, and release more significant potential for economic growth.

Since the existing research focuses on the impact of digital inclusive finance on economic growth and ignores regional differences, this paper analyzes the heterogeneity of the effect of digital inclusive finance on economic growth from three perspectives: the level of regional economic development, the degree of development of digital inclusive finance and the level of scientific and technological innovation, and further improves the existing research results [12, 14, 52].

## Conclusions and policy implications

It is rare that the existing research incorporates digital inclusive finance, industrial structure, and economic growth into a unified analytical framework. Based on the panel data of 13 cities in Beijing, Tianjin, and Hebei from 2011 to 2020, this paper empirically studies the impact of digital inclusive finance on economic growth and innovatively analyzes the role of industrial structure in the impact of digital inclusive finance on economic growth from three dimensions: industrial structure transformation speed, industrial structure upgrading and industrial structure rationalization. According to the median economic growth level, digital inclusive finance level, and technological innovation level, the heterogeneity is compared and analyzed. Specifically, the following conclusions are drawn:

First, digital inclusive finance is positively related to economic growth. From the perspective of digital inclusive finance, the depth of use of digital inclusive finance has the strongest correlation with economic growth, followed by the breadth of coverage, and the degree of digitization is the smallest. Secondly, from the perspective of the mechanism of action, the speed of industrial structure transformation and the rationalization of industrial structure play an intermediary role in the effect of digital inclusive finance on economic growth, while the upgrading of industrial structure does not have an intermediary effect. Third, from the results of heterogeneity analysis, the impact of the economically developed group, the better digital inclusive finance group, and the more developed technological innovation group on economic growth is better than that of the corresponding economically underdeveloped group, the lower digital inclusive finance group, and the less developed technology group.

Based on the above conclusions, the following enlightenment is obtained: First, improve the breadth, practical depth, and digitization of digital inclusive financial coverage. Continuously improve the construction of digital inclusive economic infrastructure, improve network coverage and penetration of terminal equipment such as computers, promote the digitization of financial products, realize the deep integration of digital technology and financial services, and improve the accuracy of financial resource allocation. Secondly, promote the transformation of industrial structure and improve the deep integration of industrial structure and digital inclusive finance. Change the traditional rough production mode with the help of digital technology, develop new digital industries, realize high-tech industrial clusters according to the actual development of each region and reasonable layout, and optimize the efficiency of resource allocation. Finally, according to the actual situation of the region, a differentiated digital inclusive financial policy is implemented. Each region should formulate digital inclusive financial policies based on the real development of the local area. For example, in economically underdeveloped regions, we should pay attention to the construction of digital inclusive financial infrastructure, improve residents ’ financial literacy, and strengthen the publicity of digital inclusive financial knowledge. In developed regions, we should pay attention to the development of high-end products of digital inclusive finance, pay attention to the integration and development of digital inclusive finance and other industries, realize the sharing of information resources, improve the efficiency and accuracy of financial services, realize the optimal allocation of financial resources, and promote the healthy and rapid development of the economy.

This study is based on the regional research of Beijing-Tianjin-Hebei, the largest urban agglomeration in northern China and the national core growth pole, and analyzes digital inclusive finance, industrial institutions, and economic growth. Although the research views have filled in the existing theories, measuring economic growth only in quantity may be one-sided. Economic growth includes both quantitative growth and qualitative growth. Therefore, we should pay attention to the impact of digital inclusive finance on high-quality economic growth.

## Supporting information

S1 Dataset (XLSX)
